# Can Endometriosis-Related Oxidative Stress Pave the Way for New Treatment Targets?

**DOI:** 10.3390/ijms22137138

**Published:** 2021-07-01

**Authors:** Luciana Cacciottola, Jacques Donnez, Marie-Madeleine Dolmans

**Affiliations:** 1 Gynecology Research Unit, Institut de Recherche Expérimentale et Clinique, Université Catholique de Louvain, 1200 Brussels, Belgium; luciana.cacciottola@uclouvain.be; 2 Society for Research into Infertility, 1150 Brussels, Belgium; jacques.donnez@gmail.com; 3 Université Catholique de Louvain, 1200 Brussels, Belgium; 4 Department of Gynecology, Cliniques Universitaires Saint-Luc, 1200 Brussels, Belgium

**Keywords:** endometriosis, oxidative stress, iron overload, antioxidants, macrophages, hyperalgesia, chronic pain, infertility

## Abstract

Endometriosis is a disease of reproductive age characterized by chronic pelvic pain and infertility. Its pathogenesis is complex and still partially unexplained. However, there is increasing evidence of the role of chronic inflammation, immune system dysregulation, and oxidative stress in its development and progression. The latter appears to be involved in multiple aspects of the disease. Indeed, disease progression sustained by a hyperproliferative phenotype can be related to reactive oxygen species (ROS) imbalance, as numerous experiments using drugs to counteract hyperproliferation have shown in recent years. Chronic pelvic pain is also associated with cell function dysregulation favoring chronic inflammation and oxidative stress, specifically involving macrophages and mast cell activation. Moreover, there is increasing evidence of a role for ROS and impaired mitochondrial function not only as deleterious effectors of the ovarian reserve in patients with endometriomas but also in terms of oocyte quality and, hence, embryo development impairment. Targeting oxidative stress looks to be a promising strategy to both curb endometriotic lesion progression and alleviate endometriosis-associated symptoms of chronic pain and infertility. More investigations are nevertheless needed to develop effective therapeutic strategies for clinical application.

## 1. Introduction

Endometriosis is a chronic disease characterized by implantation and growth of endometrial-like tissue, including glands and stroma, outside the uterine cavity [1,2]. It affects about 10% of women of childbearing age and is associated with debilitating chronic pelvic pain and infertility [3,4], causing significant impairment to quality of life and inevitably impacting social, occupational, and psychological aspects, too [5].

Although the pathophysiology of endometriosis remains poorly understood, various biological processes are known to be key players in its development and progression. Ectopic endometrium is able to survive outside the uterus and adhere to the peritoneum by activation of adhesion molecules and their receptors [6,7] and overexpression of matrix metalloproteinases [8] and plasminogen activators [9]. These implants acquire other crucial characteristics such as unrestrained growth through steroid receptivity dysregulation [1,2,10,11], as well as proangiogenic features [12,13]. Moreover, the inflammatory response and immune system modulation promoting ectopic endometrium implantation initially, and implant maintenance and progression subsequently, also appears to be pivotal [2,14]. Oxidative stress, namely disequilibrium between production and neutralization of reactive oxygen species (ROS), has been shown to be present in these processes and looks to play a key role in disease pathogenesis and evolution [15,16,17].

As described by Nisolle and Donnez [18], there are three forms of the disease, as endometriotic lesions can develop (i) on the peritoneum as superficial disease, (ii) inside the ovaries as cysts, and (iii) as deep-infiltrating disease most commonly observed in the rectovaginal septum. Endometriosis staging is based on the anatomic location and severity of the disease according to the American Society for Reproductive Medicine (ASRM) classification [19]. While this classification has a somewhat weak correlation with pain and infertility symptoms, it does allow the comparison of surgical findings and outcomes of different treatments. Current treatment options include surgical ablation or excision of lesions in the pelvic cavity or hormone therapy to suppress lesion proliferation [1]. The latter is effective for pain and reducing the risk of recurrence after surgery but is not indicated for infertility-associated endometriosis, as hormones block ovarian function [20]. Endometriosis-related infertility can be addressed by surgery to increase the chances of pregnancy in specific cases. Furthermore, assisted reproductive technology (ART) should be offered to infertile endometriosis patients who are unsuitable candidates for natural conception [20]. Previous surgery, advanced disease stage, and association with adenomyosis and ovarian and/or deep nodular endometriosis appear to be linked to poorer ART outcomes [21,22], but in vitro fertilization (IVF) is still the most effective strategy to manage infertility in these patients [20,21,22,23].

There is a clear clinical need for new therapeutic approaches, not only to treat endometriosis-related symptoms such as chronic pain and infertility but also to curtail its recurrence and progression. Oxidative stress may be a potential therapeutic target for both objectives, as it plays a crucial role in disease development and evolution.

## 2. ROS and Antioxidant Defense

The clinical importance of oxygen toxicity was not fully appreciated until an epidemic of retrolental fibroplasia (severe retinopathy) occurred in the early 1950s [24]. Indeed, oxygen at high partial pressures is toxic to the respiratory, cardiovascular, nervous, and gastrointestinal systems. Toxicity results from the formation of ROS, which are chemically reactive molecules produced naturally within biological systems.

ROS are actually intermediaries produced by normal oxygen metabolism but are known to have deleterious effects. To protect themselves, cells have developed a wide range of antioxidant systems to limit the production of ROS, inactivate them, and repair cell damage [16,17,25,26,27,28]. In healthy individuals, ROS and antioxidants are balanced, but when the balance is tipped toward an overabundance of ROS, oxidative stress is triggered and can impact the entire reproductive lifespan of a woman, as reported by Ruder et al. [25].

It is clear that ROS are generated within cells in the course of normal cellular mechanisms and that cells are adequately equipped with a range of cytoprotective enzymes and antioxidants to combat their toxicity [16,17,25,26,28]. One of the master regulators of cytoprotective defense against oxidative stress is the nuclear factor erythroid 2-related factor 2/Kelch ECH associated protein 1 (Nrf2/KEP1) pathway [29]. Activation and translocation to the nucleus of Nrf2 initiates the transcription of a number of genes, known as antioxidant response elements, involved in redox homeostasis and protection from oxidative stress-related injury [30]. This response includes an increase in antioxidant systems such as superoxide dismutase (SOD) and catalase (CAT), elevated autophagic cell activity for repair of various cell compartments, and also mitochondrial biogenesis [29,30].

However, excessive release of ROS induces cellular damage and likely alters cellular function by regulating protein activity and gene expression. Indeed, ROS play an essential role in regulating the transcription factor nuclear factor kappa B (NFkB), which has been implicated in endometriosis [2,31]. This transcription factor triggers the expression of multiple genes encoding proinflammatory cytokines, growth and angiogenic factors, adhesion molecules, and inducible enzymes nitric oxide synthase (NOS) and cyclooxygenase (COX) [32,33]. All these constituents are expressed by activated peritoneal macrophages and are involved in the pathogenesis of endometriosis by inducing endometrial fragment adhesion, proliferation, and neovascularization [32,34].

## 3. Origin of Oxidative Stress in the Peritoneal Cavity

### 3.1. Erythrocytes and Hemoglobin

Understanding the role of hemoglobin (Hb), heme, and the iron-induced redox balance in endometriosis has given rise to several hypotheses to explain why oxidative stress is triggered in the case of pelvic endometriosis [17] and is potentially involved in its pathophysiology. Erythrocytes, apoptotic endometrial tissue, and cell debris swept into the peritoneal cavity by menstrual reflux and macrophages have all been suggested as possible inducers of oxidative stress [16,35,36,37,38,39,40].

Erythrocytes are likely to release pro-oxidant and proinflammatory factors such as Hb and its highly toxic by-products heme and iron into the peritoneal environment. While iron and heme are fundamental to living cells, unless they are properly chelated, free iron and (to a lesser degree) heme play key roles in the formation of deleterious ROS [40,41,42,43].

**Figure 1:**
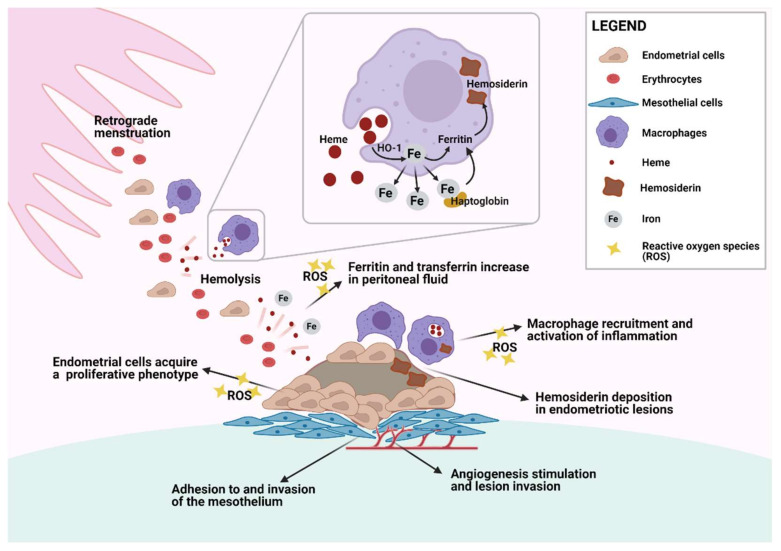
Endometrial cell and macrophage interaction in the pelvic cavity. Erythrocytes and endometrial cells are carried into the pelvic cavity by retrograde menstruation and are phagocytosed by peritoneal macrophages. Heme digestion by heme oxygenase 1 (HO-1) releases iron, which is either stored in the form of ferritin and hemosiderin or released to bind to transferrin. Endometrial cells with adhesive characteristics start to invade the mesothelium and trigger inflammatory signals that recruit more peritoneal macrophages. Local inflammation and increased levels of reactive oxygen species (ROS) contribute to the acquisition of a proliferative phenotype and proangiogenic features that are crucial to endometriotic lesion development. Created with biorender.com (accessed on 29 May 2021).

However, erythrocytes are observed in the peritoneal cavity of 90% of menstruating women [18], so it is puzzling why some patients develop macroscopically visible peritoneal endometriotic lesions while others do not [44]. One theory is that peritoneal protective mechanisms may be swamped by menstrual reflux in some patients, either because of its abundance or due to defective scavenging systems [40,41,42,43] (Figure 1).

### 3.2. Iron Metabolism in the Pelvic Cavity

In case of hemorrhage, lysis of erythrocytes results in iron overload, which in turn causes iron-mediated damage, oxidative injury, and inflammation [45,46,47], so iron may well be implicated in endometriosis development [28,43,45,47]. A crucial defense mechanism counteracting the effects of hemorrhage is mediated by haptoglobin (Hp), which binds to extracellular Hb, thereby weakening its oxidative and inflammatory potential. Moreover, Hp promotes the clearance of Hb via the CD163 scavenger receptor present on macrophages [46].

As in most tissue, activated macrophages recruited inside the pelvic cavity of women play a vital role in the degradation of erythrocytes, as indicated by the presence of numerous iron-laden macrophages in the peritoneal fluid of endometriosis subjects [33,34] and mice injected intraperitoneally with erythrocytes [40]. Macrophages generally phagocytose senescent erythrocytes or endocytose the Hb–Hp complex [46]. Thus, Hb and heme metabolism by heme oxygenase (HO) releases iron, which is then integrated into ferritin in macrophages or returned to the iron transporter transferrin via peritoneal fluid [48].

Iron conglomerates have also been encountered in endometriotic lesions [37,43], composed of hemosiderin, another iron storage form found in conditions of iron overload and usually associated with toxic pathological states in humans. Iron storage is significantly greater in peritoneal macrophages of endometriosis patients than in controls [43]. Cellular iron storage within ferritin limits the ability of iron to generate ROS, conferring an antioxidant effect [43]. However, continued delivery of iron to macrophages may overwhelm the capacity of ferritin to store and sequester the metal, causing oxidative damage to cells.

### 3.3. Cellular Damage and Adhesion and Growth of Ectopic Endometrial Tissue

In many other tissues, iron is known to induce oxidative stress, resulting in macromolecular oxidative damage, tissue injury, and chronic inflammation [39,47,49]. It was therefore suggested that oxidative stress could be responsible for local destruction of the peritoneal mesothelium, creating adhesion sites for ectopic endometrial cells [16,33,50] and promoting invasion [15,51,52].

### 3.4. Heme Oxygenase-1 Detoxification System

Heme oxygenase-1 (HO-1) is a heme-degrading enzyme strongly upregulated by heme that shields cells from heme-induced oxidative stress by generating beneficial molecules such as carbon monoxide (CO), bilirubin, and ferritin. Indeed, induction of HO-1 is accompanied by increased ferritin synthesis, scavenging of free iron, and, ultimately, protection against its adverse effects [53]. Bilirubin is an important antioxidant defending against oxidative damage and inflammation [54], while CO is a soluble gas acting as a signal molecule.

HO-1, which has a number of triggers such as ROS, free heme, heavy metals, and cytokines [28,55], is able to break down Hb and release iron from heme. Together with iron, HO-1 boosts levels of CO and biliverdin, both of which play a unique protective and antioxidant role, as well as possessing anti-inflammatory and antiapoptotic properties. HO-1-induced cytoprotective effects require co-expression of ferritin, but CO has been shown to exert significant cytoprotective, anti-inflammatory, and antiapoptotic properties all by itself [28,36].

In endometriosis patients, Hb concentrations were found to be higher in peritoneal fluid, and stronger HO expression was observed in ectopic endometrium, particularly red lesions, compared to eutopic endometrium and mesothelial cells [16,36]. However, since inducible HO-1 was weakly expressed by macrophages and mesothelial cells that make up the majority of cells in the peritoneal cavity, and because there was no concomitant upturn in peritoneal fluid levels of its final by-product bilirubin, it strongly infers that detoxifying systems, while present, may be inadequate to metabolize Hb in the case of endometriosis [37,43].

In conclusion, oxidative stress arises when the balance between ROS production and antioxidant defense is disrupted [16,26,35,52] due to either insufficient antioxidant protection or excess ROS production.

## 4. ROS as Potential Therapeutic Targets for Endometriosis Progression

### 4.1. Proliferative Phenotype

A proliferative phenotype of endometrial cells in patients affected by endometriosis compared with unaffected subjects has been identified, showing features that are commonly observed in cancer cells: uncontrolled growth, proangiogenic factors, and the ability to escape apoptosis [56]. A proliferative phenotype is sustained by dysregulation of estrogen and progesterone receptor expression in ectopic endometrium, leading to progesterone resistance and curtailing the capacity of progestins to control endometriosis progression [2,57]. Such hormonal dysregulation enhances the inflammatory environment through prostaglandin generation and contributes to pro-oxidant conditions in ectopic lesions [2]. For this reason, therapeutic strategies combining non-steroidal anti-inflammatory drugs with chronic hormone treatment, such as estroprogestins or progestins alone [57,58], are effective in managing disease progression only in two-thirds of cases [57]. Selective progesterone receptor modulators (SPRMs), such as mifepristone, are also able to significantly reduce endometrial proliferation and, hence, the development of endometriotic lesions [59]. However, their pharmacological safety profile, showing increased toxicity and risk of hepatic dysfunction with chronic use, has limited their application in clinical settings and further research into their suitability [59].

**Figure 2:**
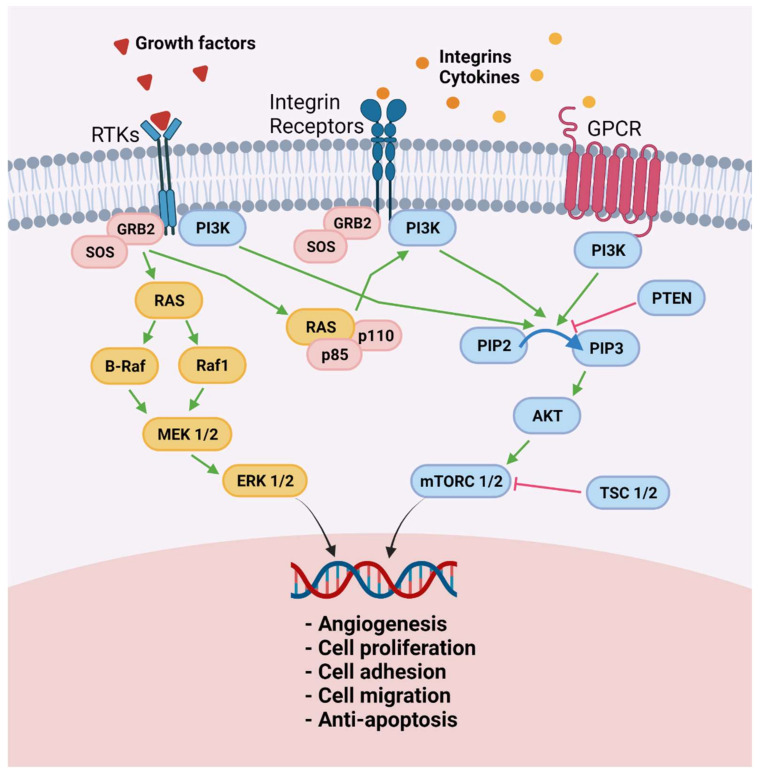
Raf/MEK/ERK and PI3K/Akt/mTOR pathways in ectopic endometrial cells. Molecule-like growth factors, integrins, and cytokines that bind to receptors on the cell surface activate both the Raf/MEK/ERK and the PI3k/Akt/mTOR signaling pathways in endometrial cells to acquire proangiogenic features, proliferation ability, increased adhesion and migration capacity, and resistance to apoptosis: RTKs, receptor tyrosine kinases; GRB2, growth factor receptor-bound protein 2; SOS, son of sevenless; Raf, serine/threonine-protein kinase; MEK ½, mitogen-activated protein kinase; ERK ½, extracellular signal-regulated kinase; GPCR, G protein-coupled receptor; PI3K, phosphoinositide 3 kinase; PTEN, phosphatase and tensin homolog; p110; p85 PIP2, phosphatidylinositol 4,5-bisphosphate; PIP3, phosphatidylinositol 3,4,5-trisphosphate; Akt, protein kinase B; mTORC1, mammalian target of rapamycin complex 1; TSC ½, tuberous sclerosis complex 2. Created with biorender.com (accessed on 28 May 2021).

Increased ROS levels in endometriosis are not only a consequence of chronic inflammation that characterizes this disease but are also caused by ROS detoxification pathway dysregulation [60]. Both an upturn in hydrogen peroxide production by mitochondrial superoxide dismutase activity and a drop in catalase activity were found to elevate ROS levels in endometriotic lesions [60]. ROS may therefore play a role as signaling molecules to sustain an endometriosis-associated proliferative phenotype. Indeed, they are known to exert influence as second messengers for cell proliferation by activating growth-related signaling pathways such as serine/threonine-protein kinase/mitogen-activated kinase/extracellular signal-regulated kinase (Raf/MEK/ERK), which is involved in proliferation in response to higher endogenous ROS levels [61] (Figure 2). ERK 1 and 2, members of the MAPK family, reside in the cytoplasm and undergo nuclear translocation when triggered by MEK 1 and 2 to promote gene expression for cellular proliferation, survival, differentiation, and adhesion. This pathway was found to be upregulated in both eutopic and ectopic endometrial cells of subjects with endometriosis [62], and its upregulation was related to increased ROS values [60], as also observed in cancer cells [61]. This was evidenced in both ectopic and eutopic endometrial cells, suggesting the presence of pre-existing alterations to redox balance and the Raf/MEK/ERK pathway in favor of proliferative cell phenotypes for migration and implantation of ectopic endometriotic tissue [63,64]. Downregulation of ERK1/2 activation through first-generation MEK-1/2 inhibitors demonstrated a decrease in human endometrial cell proliferation in vitro and in human endometriotic lesions grafted to nude mice [64]. Other drugs suppressing the same pathway were also tested on murine models of endometriosis (Table 1).

Leflunomide, acting as a tyrosine kinase inhibitor to suppress NFκB transcription [79], was shown to decrease proliferative activity in ectopic endometrial cells [64,80]. Sorafenib, a multikinase inhibitor targeting the serine/threonine RAF kinases (RAF-1 and B-RAF) and vascular endothelial growth factors (VEGF) receptors with antiproliferative effects in cancer [81], was found to reduce the proliferation of human stromal cells in deep endometriotic nodules, as well as in vivo proliferation in induced murine models (with the same deep nodular endometriosis), by suppressing ERK phosphorylation and VEGF receptor 2 activity [78]. However, the use of sorafenib was shown to yield conflicting results in terms of antiproliferative and antiangiogenic properties in rat models of endometriosis [76,77]. This disparity in effectiveness may be due not only to bias from the use of animal models for endometriosis but also from variations in stromal and endometrial cell regulators in endometriotic lesions. Indeed, ectopic stromal cell proliferation was found to be less related to ROS-dependent ERK1/2 upregulation, as in the case of endometriomas, suggesting other mechanisms maintaining cells in a hyperproliferative state [69]. Among them, the phosphoinositol 3-kinase/protein kinase B/mammalian target of rapamycin (PI3K/Akt/mTOR) pathway, known to regulate numerous cell functions such as metabolism, growth, and survival, may play a key role since it was shown to be activated in both ovarian and deep nodular endometriosis [82] (Figure 2).

Cannabinoid agonists have antiproliferative properties inhibiting both the Raf/MEK/ERK and the PI3K/Akt/mTOR pathways and have proved effective in suppressing proliferation in deep nodular endometriosis [65], confirming the hypothesis of multiple proliferative pathways responsible for endometriotic lesion progression. Moreover, the drop in proliferation rate in cells isolated from deep-infiltrating endometriotic lesions after treatment with temsirolimus, an inhibitor of the PI3K/Akt pathway through its effector mTOR, evidenced the specific link between activation of this signaling pathway and the hyperproliferative phenotype of deep nodular endometriosis [69].

Although efforts made to elucidate the role of a proliferative phenotype in endometriosis have shed light on various signaling pathways involved, none of the aforementioned drugs can be considered a valid therapeutic option for endometriosis patients. Indeed, some of the cited drugs with antiproliferative and antiangiogenic impacts are approved in humans for oncological indications, but the high risk of side effects and their incompatibility with reproductive age and conception chances make them unsuitable candidates for endometriosis treatment in young patients.

Various phytochemicals, including resveratrol and naringenin, have also been tested in endometriosis models. Suppression of lesion growth was observed in preclinical studies with resveratrol thanks to its antiproliferative and antiangiogenic properties [74,75]. Naringenin is an antioxidant drug with antiproliferative and antioxidant effects. It acts by curbing Nrf2/KEAP1 pathway activation, decreasing cytoprotection from ROS, and favoring apoptosis. A reduction in endometriotic lesions through modulation of this pathway has been demonstrated in a rat model of endometriosis [70,71].

Other promising antioxidants with a safe pharmacological profile can be extracted from plants. Among them, curcumin has been widely tested in numerous pathological conditions for its anticancer, anti-inflammatory, and antioxidant properties [83]. Studies in rodents have proved the ability of curcumin to reduce endometriotic lesions through its antiangiogenic [66] and proapoptotic action [67]. However, the same characteristics were not confirmed in other experimental models using human endometrial cells [83,84]. Such divergent outcomes may be explained by low curcumin bioavailability and rapid metabolism in humans [83]. Further investigations are needed to determine whether this molecule, associated with pharmacological strategies such as liposomes or nanoparticles to enhance its bioavailability, could be a good candidate for neoadjuvant antioxidant therapy in patients with endometriosis.

Inhibiting oxidative stress with an impact on the proliferative phenotype was also attempted using N-acetylcysteine (NAC), which is not only known as an antioxidant drug but has also been shown to exert antiproliferative effects on cancer cells of epithelial origin [85]. NAC demonstrated antioxidant and antiproliferative activity in animal models of endometriosis by downregulating ERK1/2 kinase activity [72]. NAC treatment was then tried on women diagnosed with ovarian endometriosis and yielded a significant reduction in endometriotic cyst volume and endometriosis-related pain [73].

### 4.2. Iron Overload

The presence of iron conglomerates in endometriotic lesions inducing oxidative stress in the pelvic cavity may play a crucial role not only in endometriosis pathogenesis but also in disease progression [40,41,42,43]. Iron overload activates proinflammatory signaling, such as NFkB and interleukin (IL)-1β, and triggers proliferation through cell cycle progression [86,87]. Iron chelators have been shown to mitigate iron overload effects, including excessive proliferation, serving as potential treatment options for cancer therapy [88,89] (Table 1). Among iron chelators, desferrioxamine was able to reduce iron deposits and decrease the proliferation rate of glandular cells in endometriotic lesions in a murine model [40]. Iron overload in endometriosis was also targeted by erastin, a molecule able to boost ROS levels in the presence of iron accumulation to initiate ferroptosis, a cell death mechanism based on lethal peroxidation accumulation [90]. Erastin was found to induce ferroptosis in human endometrioma-derived cells in vitro and reduce the volume of endometriotic lesions in a murine model [68].

## 5. Treatment of Chronic Pain by Decreasing Macrophage Activity

The innate immune response is considered a central player in the pathophysiology of endometriosis since it involves proliferation and vascularization signaling in lesions [2,91]. In the presence of pelvic endometriosis, macrophage activation in the peritoneal cavity might promote oxidative stress, generating lipids and various proteins, including low-density lipoprotein peroxidation [15,91]. Non-neuronal cells such as mast cells and macrophages play an active role in pain in ectopic endometrium, through the release of proinflammatory cytokines such as tumor necrosis factor α (TNF-α) and IL-1β, and growth factors such as insulin growth factor 1 (IGF-1), resulting in nerve fiber recruitment and neuroinflammation [92,93,94,95,96]. These are key mechanisms for the development and maintenance of chronic pain [97]. Indeed, repeated stimuli lead to the enhanced responsiveness of pain nerve fibers, with an increase in their excitability, causing pain hypersensitivity and endometriotic lesion-related allodynia [98,99].

Based on the hypothesis that chronic inflammation plays a key role in endometriosis progression and associated hyperalgesia through macrophage activation, numerous experiments have been conducted in attempts to modulate the immune response in endometriosis models. While immune response modulation by administration of T-cell regulatory drugs such as IL-2 and interferon α (IFNα) did not have any impact on endometriotic lesions in clinical trials [100,101], strategies able to regulate upstream triggers of the immune response, such as macrophages and mast cells, have yielded promising results (Table 2).

Peritoneal macrophage depletion was achieved using delivery systems such as liposomal bisphosphonate [102] and liposomal clodronate [96], which ensure drug delivery to phagocytic cells while minimizing toxicity in non-phagocytic cells. A decrease in endometriotic implants and systemic production of proinflammatory cytokines were observed with this therapeutic strategy in induced endometriosis in mice [96,102]. The role of IGF-1 in macrophage polarization and neurogenic activity as a nerve-sensitizing factor in an endometriosis-related inflammatory environment was also explored. Activated macrophage inhibition was achieved by the administration of linsitinib, an IGF-1 receptor inhibitor, resulting in endometriosis-associated pain alleviation and reduced nerve growth [96].

Some drugs act as mast cell stabilizers, reducing their activation and degranulation of proinflammatory cytokines. A number of mast cell stabilizers, such as leukotriene receptor antagonists [103] and ultramicronized palmitoylethanolamide (PEA) [107], have been tested on endometriosis models in preclinical studies. They had a positive impact on chronic pain through a significant reduction in mast cell degranulation and release of cytokines such as TNF-α, but there was no decrease in endometriotic lesion size [108]. Clinical studies on PEA combined with polydatin, another mast cell stabilizer, do suggest some effect on endometriosis-related pain [108], but further studies need to validate this therapeutic approach prior to introduction into clinical practice [95,109].

Some antioxidants with pleiotropic effects on neuroinflammation have also been considered as potential therapeutic options. Among them, melatonin is an antioxidant and anti-inflammatory drug able to modulate levels of several cytokines [110], including brain-derived neurotrophic factor (BDNF), a neuromediator playing a central role in hyperalgesia sensitization, and hence in chronic pain [111,112]. Melatonin modulation of BDNF may be of special interest in endometriosis, as it is partially dependent on estrogen variations and steroidogenesis [106,111]. Studies in animal models have shown melatonin to induce regression and atrophy of endometriotic lesions [104,105]. Administration of melatonin for 8 weeks also proved effective at reducing discomfort in patients with endometriosis and suffering chronic pelvic pain [106].

Although further investigations need to establish whether targeting macrophages is a valid therapeutic option for endometriosis treatment, preliminary results look promising, possibly involving a role for such a strategy in slowing disease progression and relieving chronic pain-related symptomatology.

## 6. Targeting Oxidative Stress to Treat Endometriosis-Related Infertility

**Figure 3:**
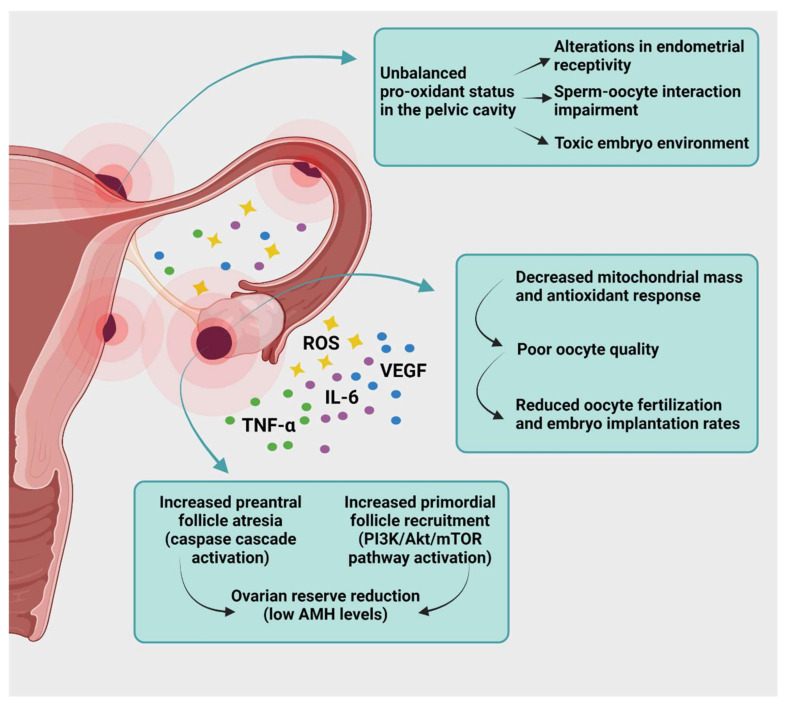
Endometriosis and infertility. Impact of ROS and inflammatory cytokines on the reproductive tract of patients with pelvic and ovarian endometriosis. Endometriosis affects the ovarian reserve and quality of oocytes and contributes to a toxic pelvic environment, reducing the chances of fertilization and implantation: ROS, reactive oxygen species; TNF-α, tumor necrosis factor α; IL-6, interleukin 6; PI3K/Akt/mTOR, phosphoinositol 3-kinase/protein kinase B/mammalian target of rapamycin; AMH, anti-Müllerian hormone. Created with biorender.com (accessed on 28 May 2021).

Endometriosis may affect fertility potential in numerous ways. Pelvic anatomy alterations due to adhesions and tubal dysfunction, a diminished ovarian reserve with lower oocyte and embryo quality, along with decreased implantation and pregnancy rates because of endometrial receptivity alterations, have been widely investigated as a cause of endometriosis-related infertility [20,113] (Figure 3).

The ovarian reserve, namely the number of primordial follicles that are quiescent in the cortex, may be negatively impacted by surgical ablation of endometriomas, with a significant drop in anti-Müllerian hormone (AMH) levels [114,115]. However, the ovarian reserve has been found to be reduced in subjects even before surgery [115], and its accelerated depletion is associated with the presence of endometriomas and fibrosis in cortical tissue, the latter being the consequence of both chronic inflammation and oxidative stress [116]. Increased follicle recruitment and atresia were indeed observed in the ovarian cortex close to endometriomas compared with the contralateral ovary [117], resulting in focal exhaustion of early-stage follicles. Numerous cytokines and molecules that favor ectopic lesion hyperproliferation, such as VEGF, IL-6, and TNF-α, may support accelerated follicle activation in the surrounding healthy ovarian cortex, as well as triggering the PI3K/Akt/mTOR signaling pathway involved in primordial follicle recruitment [118]. Moreover, ROS can directly modulate the ovarian reserve, not only by enhancing the proinflammatory environment and PI3K/Akt/mTOR pathway activation [119,120] but also by precipitating cell death due to cumulative DNA damage [121,122]. Intraovarian oxidative stress is further compounded around endometriomas by the presence of increased uncoupled iron levels resulting from the imbalance between iron storage mechanisms and its reactivity [123].

A reduced ovarian reserve due to the presence of endometriomas or as a consequence of their surgical ablation only partially explains poor IVF results in women with endometriosis. Indeed, recently published data on IVF outcomes revealed lower oocyte survival, embryo development, and clinical pregnancy rates in younger (<35 years) endometriosis patients who had undergone fertility preservation by oocyte vitrification, compared with those undergoing elective fertility preservation [124]. This suggests that impaired oocyte quality and implantation may well play an important role in the pathogenesis of endometriosis-related infertility.

Oocyte quality depends on mitochondrial function and enzymatic setting to balance intracellular ROS levels [125]. Mature oocytes from women with endometriosis are characterized by more abnormal mitochondria, decreased mitochondrial mass [126,127], and differing expression of patterns regulating antioxidant response [128]. These aspects are crucial to fertility potential, as impaired mitochondrial function may impact energy production and lead to oocyte arrest and degeneration during the fertilization process.

The other key element is endometrial receptivity due to endometriosis-sustained chronic pelvic inflammation [129]. Peritoneal fluid from women with endometriosis has pro-oxidant status due to an imbalance between ROS generation and antioxidant defense systems, resulting in less efficient protection of oocytes. Antioxidants such as vitamin C, vitamin E, thioredoxin, SOD, and glutathione have been found to be lower in follicular fluid from infertile patients with endometriosis than in women with non-endometriosis-related infertility [130,131,132,133]. Despite robust evidence showing altered redox balance in the pelvic and intrafollicular environment in the case of endometriosis, supplementation with immunomodulators able to decrease inflammation and oxidative stress, such as pentoxifylline [134], or indeed antioxidants such as vitamin C and E [135,136], failed to yield any advantage in terms of pregnancy rates.

## 7. Conclusions

Recent literature on the subject supports the notion that oxidative stress plays a key role in the pathogenesis of endometriosis and its evolution and symptom presentation. The main symptoms of endometriosis, namely chronic pain and infertility, are contingent on the establishment of chronic inflammation, with dysregulation of the immune system and multiple cell functions, including the control of ROS generation. Targeting oxidative stress could prove a winning strategy to manage both endometriotic lesion progression by curbing the hyperproliferative phenotype acquired by endometrial cells and inhibiting iron overload, and also endometriosis-associated symptoms. Chronic pain and hyperalgesia may be mitigated by innate immune response modulation, focusing particularly on macrophages and mast cells. Regarding infertility, there is growing evidence that oxidative stress is involved in accelerated follicle depletion, poor oocyte quality, and lower fertilization rates. Our increased understanding will undoubtedly lead to improved treatments in the near future, thanks to novel therapeutic strategies targeting ROS imbalance and impaired mitochondrial function.

## Figures and Tables

**Table 1:** Drugs antagonizing the hyperproliferative phenotype.

Reference	Drug	Effect	Endometriosis Source	Experimental Model
Leconte et al., 2010 [65]	Cannabinoid agonists	Inhibition of PI3K/Akt/mTOR pathway	Human (DIE)	In vitro culture; xenotransplantation murine model
		Drop in cell proliferation		
		Reduced ROS generation		
		Lower NO levels		
		Decline in endometriotic lesion volume		
Zhang et al., 2011 [66]	Curcumin	Decline in endometriotic lesion volume	Rodent (rat)	Autotransplantation rat model
		Lower VEGF levels		
		Lower microvessel density		
Jana et al., 2012 [67]	Curcumin	Increase in apoptosis rates	Rodent (mouse)	Autotransplantation murine model
		Higher MMP-3 levels		
Defrère et al., 2006 [40]	Desferrioxamine	Drop in cell proliferation	Human (menstrual endometrium)	Xenotransplantation murine model
		lower iron levels in lesions, macrophages, and peritoneal fluid		
Li et al., 2020 [68]	Erastin	Increase in total and lipid ROS values	Human (endometriomas)	Xenotransplantation murine model
		Upturn in iron levels		
		Decline in endometriotic lesion volume		
Leconte et al., 2011 [69]	ERK inhibitors	Inhibition of Raf/MEK/ERK pathway	Human (DIE)	In vitro culture; xenotransplantation murine model
	Temsirolimus	Drop in cell proliferation		
		Inhibition of PI3K/Akt/mTOR pathway		
	NAC	Drop in cell proliferation		
		Reduced ROS generation		
Ngo et al., 2010 [64]	Leflunomide, MEK 1/2 inhibitors	Inhibition of Raf/MEK/ERK pathway	Human (endometriomas and DIE)	In vitro culture; xenotransplantation murine model
		Drop in cell proliferation		
		Decline in endometriotic lesion volume		
Park et al., 2017 [70]	Naringenin	Drop in cell proliferation	Human (pelvic endometriotic lesions)	In vitro culture
		Inhibition of PI3K/Akt/mTOR pathway		
		Increased levels of ER stress and ROS		
Kapoor et al., 2018 [71]	Naringenin	Decline in endometriotic lesion volume	Rodent (rat)	In vitro culture; autotransplantation rat model
		Lower TNFα and NO levels		
		Inhibition of Nrf2/KEAP1 pathway		
		Increase in mitochondrial damage, ROS, and apoptosis		
Pittalunga et al., 2010 [72]	NAC	Decline in endometriotic lesion volume	Rodent (mouse)	Allotransplantation murine model
		Decrease in COX-2 and MMP-9 expression		
Porpora et al., 2013 [73]	NAC	Decline in endometriotic lesion volume	Human (endometriomas >3 cm)	Endometriosis patients with chronic pain and infertility
		Decrease in COX-2 expression		
		Alleviation of endometriosis-related pain		
Rudzitis-Auth et al., 2013 [74]	Resveratrol	Drop in cell proliferation	Rodent (mouse)	Allotransplantation murine model
		Decline in endometriotic lesion volume		
		Lower microvessel density		
Taguchi et al., 2014 [75]	Resveratrol	No difference in cell proliferation	Human (endometriomas)	In vitro culture
		Decrease in IL-8 expression		
Ozer et al., 2013 [76]	Sorafenib	No difference in implant volume	Rodent (rat)	Autotransplantation rat model
		Lower microvessel density		
Yildiz et al., 2015 [77]	Sorafenib	No difference in proliferation rates	Rodent (rat)	Autotransplantation rat model
		No difference in apoptosis rates		
		Decrease in VEGF expression		
Leconte et al., 2015 [78]	Sorafenib	Inhibition of Raf/MEK/ERK pathway	Human (endometriomas and DIE)	In vitro culture; xenotransplantation murine model
		Drop in cell proliferation		

ERK, extracellular signal-regulated kinase; NAC, N-acetylcysteine; MEK, mitogen-activated protein kinase; PI3K/Akt/mTOR, phosphoinositol 3-kinase/protein kinase B/mammalian target of rapamycin; ROS, reactive oxygen species; Raf, serine/threonine-protein kinase; NO, nitric oxide; VEGF, vascular endotheliam growth factor; MMP-3/9, matrix metralloproteinase 3/9; TNF-α, tumor necrosis factor α; Nrf2/KEAP1, nuclear factor erythroid 2-related factor 2/Kelch ECH associated protein 1; ER, endoplasmic reticulum; COX-2, cyclooxygenase 2; IL-8, interleukin 8; DIE, deep-infiltrating endometriosis.

**Table 2:** Drugs decreasing macrophage and mast cell infiltration and activation.

Reference	Drug	Effect	Endometriosis Source	Experimental Model
Haber et al., 2009 [102]	Liposomal bisphosphonate	Decline in endometriotic lesion volume	Rodent (rat)	Autotransplantation rat model
		less macrophage infiltration		
Foster et al., 2019 [96]	Liposomal clodronate	Less macrophage infiltration	Human (pelvic endometriotic lesions)	In vitro culture; xenotransplantation murine model
		Decrease in IGF-1 expression		
	Linsitinib	Attenuation of hyperalgesia		
Ihara et al., 2004 [103]	Leukotriene receptor antagonists	Lower stromal proliferation rates	Rodent (rat)	Autotransplantation rat model
		Decline in mast cell infiltration and activation		
Guney et al., 2008 [104]	Melatonin	Decline in endometriotic lesion volume	Rodent (rat)	Autotransplantation rat model
		Increased SOD and CAT activity		
		Lower MDA levels		
Yildirim et al., 2009 [105]	Melatonin	Decline in endometriotic lesion volume	Rodent (rat)	Autotransplantation rat model
		Increased SOD and CAT activity		
Schwertner et al., 2013 [106]	Melatonin	Alleviation of chronic pain	Human (pelvic endometriotic lesions)	Endometriosis patients with chronic pain
		Improvement in dysmenorrhea and dyspareunia		
		Improvement in dyschezia and dysuria		
		Lower BDNF levels		
Iuvone et al., 2016 [107]	PEA	Decrease in mast cell numbers	Rodent (rat)	Autotransplantation rat model
		Decline in endometriotic lesion volume		
Idraccolo et al., 2017 [108]	PEA + polydatin	Alleviation of chronic pain	Human (pelvic endometriotic lesions)	Endometriosis patients with chronic pain
		Improvement in dysmenorrhea and dyspareunia		
		No improvement in dyschezia		

PEA, ultramicronized palmitoylethanolamide; IGF-1, insulin growth factor 1; SOD, superoxide dismutase; CAT, catalase; MDA, malondialdehyde; BDNF, brain-derived neurotrophic factor.
